# Child survival and BCG vaccination: a community based prospective cohort study in Uganda

**DOI:** 10.1186/s12889-015-1497-8

**Published:** 2015-02-22

**Authors:** Victoria Nankabirwa, James K Tumwine, Proscovia M Mugaba, Thorkild Tylleskär, Halvor Sommerfelt, for the PROMISE- EBF Study Group

**Affiliations:** Department of Epidemiology and Biostatics, School of Public Health, College of Health Sciences, Makerere University, Kampala, Uganda; Centre for Intervention Science in Maternal and Child Health (CISMAC), Centre for International health, University of Bergen, Bergen, Norway; Department of Paediatrics and Child Health, School of Medicine, College of Health Sciences, Makerere University, Kampala, Uganda; Centre for International Health, Department of Global Public Health and Primary Care, University of Bergen, Bergen, Norway; Department of International Public Health, Norwegian Institute of Public Health, Oslo, Norway

**Keywords:** BCG, Vaccination, Vaccines, Child, Survival, Non-specific-effects

## Abstract

**Background:**

Data on non-specific effects of BCG vaccination in well described, general population African cohorts is scanty. We report the effects of BCG vaccination on post-neonatal infant and post-infancy mortality in a cohort of children in Mbale, Eastern Uganda.

**Methods:**

A community-based prospective cohort study was conducted between January 2006 and February 2014. A total of 819 eligible pregnant women were followed up for pregnancy outcomes and survival of their children up to 5 years of age. Data on the children’s BCG vaccination status was collected from child health cards at multiple visits between 3 weeks and 7 years of age. Data was also collected on mothers’ residence, age, parity, household income, self-reported HIV status as well as place of birth. Multivariable Cox proportional hazards regression models taking into account potential confounders were used to estimate the association between BCG vaccination and child survival.

**Results:**

The neonatal mortality risk was 22 (95% CI: 13, 35), post-neonatal infant mortality 21 (12, 34) per 1,000 live births and the mortality risk among children between 1 and 5 years of age (post-infancy) was 63 (47, 82) per 1,000 live births. The median age at BCG vaccination was 4 days. Out of 819 children, 647 (79%) had received the BCG vaccine by 24 weeks of age. In the adjusted analysis, the rate of post-neonatal death among infants vaccinated with BCG tended to be nearly half of that among those who had not received the vaccine (adjusted HR: 0.47; 95% CI: 0.14, 1.53). BCG vaccination was associated with a lower rate of death among children between 1 and 5 years of age (adjusted HR: 0.26; 95% CI: 0.14, 0.48).

**Conclusion:**

The risk of early childhood death in Mbale, Uganda is unacceptably high. BCG vaccination was associated with an increased likelihood of child survival.

## Background

An estimated 145,000 children under five years of age die each year in Uganda [1] and the country’s under-five mortality is estimated to be 90 per 1,000 live births [2]. Of these deaths, 63% occur in the first year of life. Under-five mortality in Uganda has dropped between 1990 and 2012 with an average annual rate of 1.9% [3]. To meet the fourth Millennium Development Goal of reducing this mortality by two thirds from 147/1,000 in 1990 to 49/1,000 in 2015, an even greater reduction in the number of child deaths is necessary. Achievement of this goal depends in part on the identification and implementation of effective child survival interventions [4], particularly vaccination against common childhood diseases like diphtheria, tetanus, polio, measles, pneumonia and tuberculosis (TB). In Uganda, children receive the BCG vaccine at birth or at the first contact with the health service, primarily to protect them against severe forms of TB. Studies from Guinea Bissau, Senegal, and Malawi now indicate that this vaccine may have non-specific effects on childhood morbidity and mortality that cannot be solely explained by its protective effect against tuberculosis [5-9]. It appears that children vaccinated with BCG are less likely to have septicaemia [10], acute lower respiratory tract infections and respiratory syncytial virus infections [11]. It is posited that the BCG vaccine induces its non-specific effects via epigenetic reprogramming of the innate immune cells [12]. A study among human volunteers showed that with BCG vaccination, the production of IFN-γ increased 4–7 times while the release of cytokines particularly TNF and IL-1β doubled, in response to non-tuberculous bacterial and fungal pathogens [12]. With the incidence of childhood TB declining in Sub-Saharan Africa, the importance of these probable non-specific effects is growing.

Yet, the literature supporting these non-specific effects is scanty and controversial [5]. It has been suggested that methodological challenges arising from uncontrolled confounding [13,14] and possibly survival bias in retrospective studies [13,15-17] could explain the observed association between BCG vaccination and child survival. Routine childhood vaccination, particularly BCG vaccination is a key child survival intervention. Understanding this predictor and other local predictors of child survival is important for child health programmes. But, lack of vital event registration systems in many low and middle income countries (LMICs), including Uganda, makes this difficult [18-20]. The objective of this prospective community-based cohort study was to estimate the effect of BCG vaccination on the risk of death among children under five years of age in Mbale, Eastern Uganda.

## Methods

The study was undertaken as part of the cluster-randomized PROMISE EBF intervention trial, where we promoted exclusive breastfeeding (EBF) by individual peer counselling in the intervention areas [21]. Data collection started in Uganda in January 2006, continued beyond that of the PROMISE-EBF trial, and ended in February 2014. We have previously reported on the perinatal mortality and morbidity during infancy in this cohort [22].

### Study site

The study was conducted in Mbale district, 300 km North-East of Kampala with an estimated population of 720,000, at the time of study commencement in 2006 [23]. The study area is served by Mbale Regional Referral Hospital, which double as the district and regional referral hospital. The HIV prevalence among pregnant women in antenatal clinics in Mbale was approximately 5% during the study period. Most of the people living in the area were subsistence farmers. The study was conducted in the two biggest of the 7 counties in Mbale district, namely Bungokho County (rural) and Mbale Municipality (urban). Twenty four clusters were included in the study, 18 rural and 6 urban. Six urban clusters in Mbale municipality were selected from all its three municipal divisions. Most of the urban areas were large informal settlements (urban slums). Eighteen rural clusters in Bungokho County were chosen from eight out of its eleven sub-counties. Clusters were included if they neighboured the main road out from Mbale Municipality or were on the 1^st^ or 2^nd^ branch off the main road, had a population of at least 1,000 inhabitants and represented a social and administrative unit. Children in the study area receive vaccines on the Ugandan immunization schedule which includes BCG, DPT-HepB + Hib, polio and measles. BCG vaccine (BCG-Denmark) is administered intra-dermally, on the right upper arm at birth or at first contact (dosage 0.05 ml up to 11 months and 0.10mls after the 11 months). DPT-HepB + Hib is administered in three doses, of 0.5mls each at monthly intervals, starting at 6 weeks (or first contact after that age). Polio is administered orally, in four doses (2 drops each). The first dose is given at birth or within the first two weeks and the subsequent doses are monthly starting at 6 weeks of age. Measles is given as a single dose at nine months of age.

### Study subjects

Between January 2006 and May 2008, all pregnant women in the selected clusters were identified by study recruiters. Recruiters were mostly women representatives on the local village councils in the study area. They were well respected members of the community, with good knowledge of the local geography, community members and social structures. They regularly went door to door in order to identify new pregnancies. The pregnant women were then approached by the study team and invited to participate in the trial if they resided in the study area, were seven or more months pregnant, opted to breastfeed their infants, were not intending to leave the area during the study period, and consented to participate in the study. Of the 886 pregnant women who were identified and approached, 875 (99%) accepted to participate. Of these, 12 (1.4%) women did not meet the eligibility criteria and 28 (3.2%) relocated out of the study area after recruitment, while 16 (1.8%) women experienced a stillbirth. We analysed data for the remaining 819 women.

Ethics approval was obtained from the Makerere University Research and Ethics Committee, the Uganda National Council for Science and Technology, and from the Regional Committee for Medical and Research Ethics for Western Norway (REK VEST, approval number 05/8197).

### Data collection

At recruitment, trained data collectors fluent in the local language administered a pre-tested structured questionnaire in the local language, Lumasaaba. They collected information on the current pregnancy as well as on socio-demographic characteristics, antenatal care attendance (ANC) and marital status. After birth, the mother/child pairs were visited at weeks 3, 6, 12 and 24, between 18–24 months of age, between 3 and 5 years of age and between 5 and 7 years of age. Data on vaccination, illness and the child’s vital status was collected at each of the visits. Information on vaccination status was obtained through maternal interviews and review of child health cards. To minimize information bias, child health card data was prioritized in this study. A standard World Health Organization (WHO) verbal autopsy questionnaire that had been validated in Uganda was used to collect information for a standard algorithm determining the likely cause of death [24]. The questionnaire had both an open-ended section for reporting verbatim and a closed-ended section with filter questions.

### Definitions

We categorized marital status into three categories: ‘Married’, ‘Co-habiting’ and ‘Other’. The ‘Other’ category included women that were divorced, widowed or separated. In Uganda, it is now common to find couples living together without being formally married and we classified these as ‘Co-habiting’. Place of birth was categorized into ‘home births’, which were those that took place at home with or without a traditional birth attendant or those occurring on the way to a health facility. Health facility births were those that happened at a hospital, clinic or local maternity unit. We defined parity according to the number of previous live births. Based on the Ugandan education curriculum in which primary education is scheduled for seven years, education was grouped into ‘7 years of school or less’ and ‘more than 7 years of school’. Age was categorized into four categories (less than 20 years, 20–24, 25–30 and greater than 30 years).

We created a composite index of assets (socio-economic status) using multiple correspondence analysis (MCA). Because the MCA technique allows combination and ranking of a large number of variables into fewer variables without prejudgment, it is considered a more accurate indicator of socioeconomic status (SES) than single items such as occupation or possession of particular items [25]. Also, in comparison to principal component analysis (PCA), the MCA technique is more appropriate for discrete variables. This was important in this study because several relevant variables could only be categorical. Furthermore, unlike PCA, which clusters variables together, MCA clusters the categories within these variables together [25]. We used MCA on possession of a TV, radio, mobile phone, chair, cupboard, refrigerator, type of toilet, type of house walls as well as presence of electricity and water in the home. Scores were obtained and categorized into centiles (the poorest 20%, middle 40% and the richest 40%).

### Statistical analysis

Data was analysed with Stata version 9.2 (StataCorp LP, TX, U.S.). Continuous variables were summarized with means, standard deviations, medians, ranges and interquartile ranges while percentages were calculated for categorical variables. The primary outcomes were post-neonatal infant death and post-infancy death. We calculated their confidence limits with the exact method. The neonatal mortality risk was the proportion of deaths within the first 28 days of life, the infant mortality risk the proportion of deaths in the first year of life [26], all per 1,000 live births. Post-neonatal infant mortality risk was the proportion of deaths between one month and 1 year of age while post-infancy death risk was the proportion of deaths between 1 and 5 years of age. The main outcome variables were post-neonatal infant death and post-infancy death. The main exposure variable was BCG vaccination. Other variables included as potential confounders included maternal age, parity, mother’s education, place of birth, antenatal care attendance, marital status, residence and household wealth index.

In order to take into account variable follow up time, Cox proportional hazards models were used to estimate hazard ratios (HR) and 95% confidence intervals. In the models, BCG vaccinated children contributed person-time from the date of vaccination with BCG until death, migration or last follow-up visit while non-vaccinated children contributed person-time from birth till death, migration or last follow-up visit. The multivariable regression models included variables which, in a crude analysis, were associated with deaths yielding a P-value < 0.25. The final models were also adjusted for the child’s age in days at the time of vaccination and for the design effect of the cluster-randomized PROMISE-EBF trial. We also undertook a so called landmark analysis to thwart the effects of a possible survival bias induced by the so-called “immortal person-time” [27].

## Results

**Figure 1:**
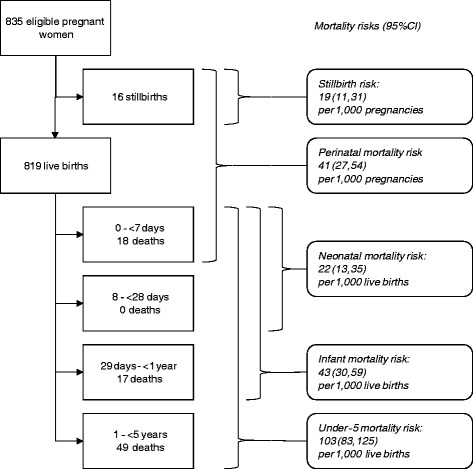
**Child mortality in a cohort of 819 children in Mbale, Eastern Uganda.**

In this cohort of 819 live born children, 83% were followed up till death or till five years of age. The mean age of the mothers was 25 years at inclusion, ranging from 14 to 44 years with an inter-quartile range (IQR) of 20 to 30 years of age. They had a mean of 7 years of formal education (range 0 to 18 years; IQR 5 to 9 years). Their mean and median parity was 3 and 2, respectively, ranging from 0 to 14, with an IQR of 3 to 5. Socio-demographic factors are presented in the tables. Most of the households, 667 (81%) were headed by men. Two hundred and fifteen women (26%) earned money for themselves. Among the women who had given birth before, 201 (25%) had lost at least one live born child. Fifty-five per cent or 452 of the women reported having been informed about HIV testing and counselling, 315 (38%) had been counselled and 251 (31%) had been tested. Out of the 156 that had been tested and knew their results, 15 (9.6%) reported to be HIV-positive. A total of 447 women out of 819 women with live births (55%) delivered at home. There were18 neonatal deaths constituting 55% of the infant deaths (Figure 1). Neonatal mortality was 22 (95% CI: 13, 35) per 1,000 live births. Conditions associated the neonatal deaths and identified via verbal autopsies were: cord prolapse, obstructed labour, antepartum haemorrhage, mal-presentations, preterm births, malaria and other causes of fever in pregnancy, neonatal tetanus and preeclampsia. None of the neonatal deaths that occurred at home were registered in the national vital registration system and there was no death certificate available for any of the deaths, including those that occurred in the health facilities. The infant mortality risk was 43 (95% CI: 30, 59) per 1,000 live births while under-five mortality was 103 (95% CI: 83, 125) per 1,000 live births.

At 6 months of age, 647 children out of a total 819 children had been vaccinated with the BCG vaccine. The median age at vaccination was 4 days, the IQR included the day of birth and day 4 after birth, and none of the vaccinated children were older than 6 months of age when they received BCG. Details on vaccination timeliness in this cohort have been presented elsewhere [28,29]. Children born at home and those living in rural areas were less likely to get vaccinated with BCG in comparison to their counterparts born at health facilities or residing in urban areas. There were no major differences between BCG vaccinated and unvaccinated children with regards to other background characteristics recorded in this study (Table 1).

**Table 1:** **Background characteristics for BCG vaccinated and BCG unvaccinated children in a cohort of 819 children in Mbale, Eastern Uganda**

	**BCG vaccination status**	
**Variable**	**Unvaccinated N = 172 n (%)**	**Vaccinated N = 647 n (%)**
Maternal age		
<20 years	39 (22.7)	142 (22.0)
20-24	51 (29.7)	212 (32.2)
25-29	47 (27.3)	150 (23.2)
≥30	35 (20.6)	143 (22.1)
Parity		
0	47 (27.3)	153 (23.7)
1-2	50 (29.1)	198 (30.6)
3-4	38 (22.1)	151 (23.3)
5 or more	37 (21.5)	145 (22.4)
Mother’s education		
>7 years	44 (25.6)	196 (30.3)
≤7 years	128 (74.4)	451 (69.7)
Residence		
Rural	118 (68.6)	517 (79.9)
Urban	54 (31.4)	130 (20.1)
Marital status		
Married	103 (59.9)	402 (62.1)
Co-habiting	47 (27.3)	193 (29.8)
Other^a^	22 (12.8)	52 (8.04)
Maternal HIV status		
Negative	31 (18.0)	110 (17.0)
Positive	5 (2.91)	10 (1.55)
Don’t know	136 (79.1)	527 (81.5)
Household wealth index		
Poorest 20%	28 (16.3)	135 (20.9)
Middle 40%	65 (37.8)	261 (40.3)
Richest 40%	79 (45.9)	251 (38.7)
Antenatal care attendance		
No	117 (68.0)	473 (73.1)
Yes	55 (32.0)	174 (26.9)
Place of birth		
Home/TBA	114 (66.3)	333 (51.5)
Hospital/clinic	58 (33.7)	314 (48.5)
Peer counselling for exclusive breastfeeding		
No	88 (51.2)	306 (47.3)
Yes	84 (48.8)	341 (52.7)

^a^Includes women that were separated, divorced or widowed.

### Post-neonatal infant deaths

The 17 post-neonatal infant deaths constituted 49% of the 33 infant deaths. This translated to a post-neonatal infant mortality risk of 21 (12, 34) per 1,000 live births in the cohort of 801 babies. Immediate causes of post-neonatal infant deaths included malaria, respiratory tract infections, and diarrhoea. In an analysis in which BCG vaccinated children contributed time from the registered date of vaccination, the rate of post-neonatal death among infants who received the BCG vaccine was half of that among those who had not been vaccinated (HR: 0.47; 95% CI: 0.14, 1.53). The landmark analysis yielded a HR of 0.76 (95% CI 0.20, 2.87). Infants whose mothers’ self-reported as HIV positive were 34 times (HR: 34.4; 95% CI: 2.91, 407.1) as likely to die in the post-neonatal period as children whose mothers’ self-reported as HIV negative (Table 2).

**Table 2:** BCG vaccination and post-neonatal infant mortality in a cohort of 801 children in Mbale, Eastern Uganda* ^***a***^

**Variable**	**No. of deaths/total**	**Deaths/1,000 (95% CI)**	**Unadjusted HR (95% CI)**	**Adjusted HR (95% CI)** ^**a**^
BCG vaccination				
Yes	12/647	19 (10, 32)	0.57(0.21, 1.52)	0.47 (0.14, 1.53)
No	5/154	32 (11, 74)		1
Maternal age				
<20 years	4/178	22 (06, 57)	1	
20-24	5/259	19 (06, 44)	0.84 (0.23, 3.12)	
25-29	4/192	21 (06, 52)	0.91 (0.23, 3.66)	
≥30	4/172	23 (06, 58)	1.31 (0.35, 4.89)	
Parity				
0	4/195	21 (06, 52)	1	
1-2	5/243	21 (07, 47)	1.04 (0.28, 3.86)	
3-4	3/185	16 (03, 47)	1.08 (0.27, 4.33)	
5 or more	5/178	28 (09, 64)	1.48 (0.40, 5.51)	
Mother’s education				
>7 years	3/233	13 (03, 37)	1	1
≤7 years	14/568	25 (14, 41)	2.05 (0.59, 7.08)	1.62 (0.36, 7.40)
Residence				
Rural	15/624	24 (14, 39)	1	1
Urban	2/177	11 (01, 40)	0.41 (0.09, 1.77)	1.26 (0.20, 7.98)
Marital status				
Married	11/494	22 (11, 39)	1	
Co-habiting	3/234	13 (03, 37)	0.52 (0.15, 1.84)	
Other^b^	3/73	41 (09, 115)	1.58 (0.45, 5.59)	
Maternal HIV status				
Negative	1/140	07 (00, 39)	1	1
Positive	2/14	143 (18, 428)	24.33 (2.12, 268.33)	34.44 (2.91, 407.06)
Don’t know	14/647	22 (12,36)	3.15 (0.42, 23.81)	2.07 (0.26, 16.78)
Household wealth index				
Poorest 20%	6/157	38 (14, 81)	1	1
Middle 40%	8/321	25 (11, 49)	0.76 (0.27, 2.14)	0.60 (0.20, 1.84)
Richest 40%	3/323	09 (02, 27)	0.25 (0.06, 1.01)	0.16 (0.03, 0.88)
Antenatal care attendance				
No	8/223	36 (16, 69)	2.00 (0.79, 5.07)	1.84 (0.63, 5.35)
Yes	9/578	16 (07, 29)	1	1
Place of birth				
Home/TBA	11/430	26 (13, 45)	1.62 (0.61, 4.32)	
Hospital/clinic	6/371	16 (06, 35)	1	
Peer counselling for exclusive breastfeeding				
No	9/387	23 (11, 44)	1.04 (0.41, 2.62)	
Yes	9/414	19 (08, 38)	1	

*CI indicates confidence interval and HR indicates hazards ratio(s) obtained from Cox proportional hazards models.

^a^The HRs are adjusted for the variables for which a HR is given as well as for age of the child in days at the time of receiving the BCG vaccine. Clustering by the randomization units in the PROMISE-EBF trial was also taken into account.

^b^Includes women that were separated, divorced or widowed.

### Post-infancy deaths

There were 784 children alive at the end of the first year and 49 of these died between 1 and 5 years of age. The mortality risk among children in this age group was 63 (95% CI: 47, 82) per 1,000 live births. BCG vaccination within the first six months was associated with better survival for this age group in both the unadjusted (HR: 0.38; 95%CI: 0.21, 0.65) and the adjusted (HR: 0.26; 95% CI: 0.14, 0.48) analysis. The landmark analysis yielded a HR of 0.33 (95% CI 0.17, 0.64). Women between 25 and 29 years of age were less likely to have a child death in this age group than women less than 20 years of age in the adjusted analysis (HR: 0.40; 95% CI: 0.16, 0.99). There was no association between maternal education and parity with post-infancy death (Table 3).

**Table 3:** BCG vaccination and death in a cohort of 784 children between 1 and 5 years age in Mbale, Eastern Uganda*

**Variable**	**No. of deaths/total**	**Deaths/1,000 (95% CI)**	**Unadjusted HR (95% CI)**	**Adjusted HR (95% CI)** ^**a**^
BCG vaccination				
Yes	29/635	46 (31, 65)	0.38(0.21, 0.65)	0.26 (0.14, 0.48)
No	20/149	134 (84, 200)		1
Maternal age				
<20 years	16/174	92 (53, 145)	1	1
20-24	15/254	59 (33, 96)	0.65 (0.33, 1.31)	0.70 (0.33, 1.47)
25-29	7/188	37 (15, 75)	0.40 (0.16, 0.96)	0.40 (0.16, 0.99)
≥30	11/168	65 (33, 114)	0.73 (0.34, 1.57)	0.69 (0.31, 1.56)
Parity				
0	11/191	58 (29, 101)	1	
1-2	17/238	71 (42, 112)	1.13 (0.54, 2.36)	
3-4	9/182	49 (23, 92)	0.78 (0.33, 1.86)	
5 or more	12/173	69 (36, 118)	1.16 (0.52, 2.59)	
Mother’s education				
>7 years	15/230	65 (37, 105)	1	
≤7 years	34/554	61 (43, 85)	0.97 (0.53, 1.77)	
Residence				
Rural	44/609	72 (53, 96)	1	1
Urban	5/175	29 (09, 65)	0.45 (0.19, 1.10)	0.51 (0.16, 1.59)
Marital status				
Married	34/483	70 (49, 97)	1	1
Co-habiting	14/231	61 (34, 100)	0.82 (0.44, 1.53)	0.93 (0.48, 1.79)
Other^b^	1/70	14 (00, 77)	0.18 (0.02, 1.32)	0.15 (0.02, 1.10)
Maternal HIV status				
Negative	8/139	58 (25,110)	1	1
Positive	2/12	167 (21, 484)	3.09 (0.66, 14.58)	1.82 (0.22, 15.14)
Don’t know	39/633	62 (44,83)	1.07 (0.50, 2.28)	1.11 (0.49, 2.51)
Household wealth index				
Poorest 20%	9/151	60 (28, 110)	1	
Middle 40%	23/313	73 (47, 108)	1.23 (0.58, 2.73)	
Richest 40%	17/320	53 (31, 84)	0.97 (0.44, 2.16)	
Antenatal care attendance				
No	16/215	74 (43, 118)	1.20 (0.66, 2.18)	
Yes	33/569	58 (40, 80)	1	
Place of birth				
Home/TBA	30/419	72 (49, 101)	1.33 (0.75, 2.36)	
Hospital/clinic	19/365	52 (32, 80)	1	
Peer counselling for exclusive breastfeeding				
No	18/378	48 (28, 74)	0.64 (0.36,1.13)	
Yes	31/406	76 (52, 107)	1	

*CI indicates confidence interval and HR indicates hazards ratio(s) obtained from Cox proportional hazards models.

^a^Model adjusted for age of the child in days and clustering to take into account the design of the PROMISE-EBF trial.

^b^includes women that were separated, divorced or widowed.

### Gender and child death

There was no association between the post-neonatal infant’s sex and death (HR for girls versus boys = 1.08; 95% CI: 0.40, 2.89). Infant gender was not an effect measure modifier of the relationship between BCG vaccination and death (p-value for heterogeneity of HRs = 0.876). Among girls, the HR was 0.52 (95% CI: 0.10, 2.79), while among boys, the HR was 0.34 (95% CI: 0.05, 2.18). Similarly, there was no association between the 1–4 year old children’s sex and death (HR: 0.94; 95% CI: 0.53, 1.69), and gender did not modify the effect of BCG on death among them (p-value for heterogeneity of HRs = 0.586). Among girls, the HR for the association between BCG vaccination and death in this group was 0.34 (95% CI: 0.14, 0.83) while among boys it was 0.22 (95% CI: 0.09, 0.52).

## Discussion

This community-based cohort study in Mbale, Eastern Uganda, presents high risks of child death and reveals that BCG vaccination in infancy is associated with survival among children 1–5 years of age. The neonatal mortality and infant mortality risk estimates of 22 and 43 were compatible with the national Demographic and Health Survey (DHS) estimates of 27 and 47 per 1,000 live births, respectively [2]. The post-neonatal infant mortality risk was 23 per 1,000 while the mortality risk among children between 1 and 5 years of age was 63 per 1,000 children.

The rate of death among 1 to 5 year old children who had received BCG by six months of age was substantially lower than among those who had not. BCG vaccine is routinely administered to children at birth to protect them against tuberculosis (TB). But several observational studies now indicate that this vaccine may have non-specific effects on childhood illness and death that extends beyond its protective effects against TB [5-9]. A prospective study of two Senegalese birth cohorts showed that children who received a combination of BCG and DPT had a lower mortality compared to those that had not [17]. Other observational studies have linked BCG vaccination and/or presence of the BCG scar with reduced incidence of acute lower respiratory tract infections [11] and septicaemia among [10] infants. These potential non-specific effects of BCG and other childhood vaccines are growing in importance especially as the incidence of TB and of other target diseases among children decrease.

Unsurprisingly, none of the deaths that occurred at home were recorded in any formal system or had a death certificate. Many of these deaths would therefore have been missed by facility-based studies and the early neonatal deaths of mothers dying during or shortly after birth could also have been missed by retrospective surveys which are the principle sources of data on mortality in low and middle income countries. This prospective study was able to capture these deaths that would have otherwise not been counted in other designs. The absence of a functioning vital event registration system highlights the need to establish a pregnancy or reproductive health registry if one is to adequately describe trends as well as predictors’ child health and survival in populations such as this one.

This was a prospective study with good ascertainment of deaths. However, there could have been an underestimation of neonatal deaths because the distinction between early neonatal deaths and stillbirths is difficult, particularly in home births where there are no skilled health workers who can identify faint signs of life [30-32]. Additionally, some stillbirths could have been misclassified as first hour deaths [31]. But, the proportion of stillbirths and early neonatal deaths in our cohort is in line with WHO estimates for East Africa [33]. Verbal autopsies were used in this study to ascertain likely causes of neonatal deaths. Though verbal autopsies are generally accepted in many countries without vital registration, their validity and sensitivity varies widely between locations [34]. In this study, we used a standard WHO verbal autopsy tool that has been validated in Uganda [24]. Because neonatal and infant deaths were few, some of our estimates are imprecise. Several relevant variables were considered and were found not to positively confound the association between BCG vaccination and child survival. In fact, adjustment for confounders strengthened rather than weakened the association between BCG vaccination and child survival. Still it is possible, that some of the association could be ascribed to confounding if children with a lower mortality were more likely to be vaccinated compared to those with a higher mortality. It was not possible to adequately adjust for the potential confounding effect of the child’s HIV status as HIV testing was not done for the children and only a quarter of the mothers were aware of their HIV status.

Vaccination data in this study was collected prospectively and validated with vaccination cards before almost all the deaths happened. Our finding is therefore unlikely to be explained by survival bias that has been reported in some retrospective studies in Africa in which child deaths were associated with missing vaccination cards that were destroyed or in other ways lost soon after the deaths [13,14,27]. Still, we undertook a landmark analysis which intends to circumvent a survival bias that artificially inflates the association between vaccination and survival [27]. The association between infant BCG vaccination and survival among 1 to 5 year old children only slightly weakened, a change that may just as well be ascribed to the non-differential misclassification inherent in this type of analysis [27]. Our analysis among the post-neonatal infants may have slightly over-estimated the death rate in the children who had not received BCG because in those that had, person-time accrual did not start at birth but when they were vaccinated. However, this possible overestimation is likely to be small as only 12% of the vaccinated children had not received BCG by the time they were 4 weeks of age and the HR was adjusted for age. Any such overestimation would not be operational in the analysis of the association between BCG vaccination and death rate among children aged 1 to 5 years because all children in the BCG vaccinated group had been vaccinated by six months of age.

This was a secondary analysis of a data from randomized trial. As such, we did not have data on all the relevant risk factors for child survival or all potential confounding factors for the association between BCG and child survival in Mbale. Importantly, nearly 50% of the children were born at home and were not weighed at birth. This limited our ability to study birth weight as a predictor of death in this cohort, or to examine its effect on the association between BCG vaccination and child survival. Only 17% of the 819 live born children migrated out of the study area and were lost to follow up before five years of age. There were no major differences in background characteristics between children lost to follow-up and those who remained in the study.

## Conclusion

The risk of childhood death remains unacceptably high and is an important public health problem in Mbale, Uganda. BCG vaccination within the first six months of life was associated with a lower incidence of death among children between 1 and 5 years of age.

### Ethical approval

Written informed consent was obtained from each study participant for the first five visits and then repeated for each follow-up visit thereafter. Ethical approval was obtained for each visit from the Makerere University Research and Ethics Committee, the Uganda National Council for Science and Technology, and from the Regional Committee for Medical and Research Ethics for Western Norway (REC WEST, approval number 05/8197).
